# Bioactive Potential of Several Actinobacteria Isolated from Microbiologically Barely Explored Desert Habitat, Saudi Arabia

**DOI:** 10.3390/biology10030235

**Published:** 2021-03-19

**Authors:** Mohammed S. Almuhayawi, Mahmoud S. M. Mohamed, Mohamed Abdel-Mawgoud, Samy Selim, Soad K. Al Jaouni, Hamada AbdElgawad

**Affiliations:** 1 Department of Microbiology and Medical Parasitology, Faculty of Medicine, King Abdulaziz University, Jeddah 21589, Saudi Arabia; 2 Department of Botany and Microbiology, Faculty of Science, Cairo University, Giza 12613, Egypt; 3 Department of Medicinal and Aromatic Plants, Desert Research Centre, Cairo 11753, Egypt; Mohamed_drc@yahoo.com; 4 Department of Clinical Laboratory Sciences, College of Applied Medical Sciences, Jouf University, Sakaka 2014, Saudi Arabia; sabdulsalam@ju.edu.sa; 5 Hematology/Pediatric Oncology, Yousef Abdulatif Jameel Scientific Chair of Prophetic Medicine Application, Faculty of Medicine, King Abdulaziz University, Jeddah 21589, Saudi Arabia; saljaouni@kau.edu.sa; 6 Integrated Molecular Plant Physiology Research, Department of Biology, University of Antwerp, 2020 Antwerp, Belgium; hamada.abdelgawad@science.bsu.edu.eg; 7 Botany and Microbiology Department, Faculty of Science, Beni‒Suef University, Beni‒Suef 62521, Egypt

**Keywords:** actinomycetes, antioxidant, leukemia, anti-inflammatory

## Abstract

**Simple Summary:**

Bioactive natural products have been regarded as promising tools for treatment of various ailments. Among natural sources, actinomycetes have been widely explored for their potential bioactivity. In this regard, the present study has focused on the phytochemical content and biological activities of several actinobacteria isolates, which were investigated for their phenolic and flavonoid content, as well as their antioxidant, antibacterial and antiprotozoal activities. The most active isolates were further investigated for their antileukemic activity, where such isolates were shown to exert cytotoxic activity against the tested cell lines, following a mechanism that might be due to the ability of the active isolate extracts to reduce cyclooxygenase and lipoxygenase activities. Overall, isolation and characterization of the active molecule from the potential actinomycetes strains will pave the way for the development of drugs against human diseases such as blood cancer.

**Abstract:**

Biomolecules from natural sources, including microbes, have been the basis of treatment of human diseases since the ancient times. Therefore, this study aimed to investigate the potential bioactivity of several actinobacteria isolates form Al-Jouf Desert, Saudi Arabia. Twenty-one actinobacterial isolates were tested for their antioxidant (flavonoids, phenolics, tocopherols and carotenoids) content, and biological activities, namely FRAP, DPPH, ABTS, SOS and XO inhibition, anti-hemolytic and anti-lipid peroxidation as well as their antibacterial and antiprotozoal activities. Accordingly, five isolates (i.e., Act 2, 12, 15, 19 and 21) were selected and their 90% ethanolic extracts were used. The phylogenetic analysis of the 16S rRNA sequences indicated that the most active isolates belong to genus *Streptomyces*. The genus *Streptomyces* has been documented as a prolific producer of biologically active secondary metabolites against different cancer types. Thus, the anti-blood cancer activity and the possible molecular mechanisms by which several *Streptomyces* species extracts inhibited the growth of different leukemia cells, i.e., HL-60, K562 and THP-1, were investigated. In general, the five active isolates showed cytotoxic activity against the tested cell lines in a dose dependent manner. Among the potent isolates, isolate Act 12 significantly decreased the cell viability and showed maximum cytotoxic activities against both HL-60 and K562 cells, while isolate Act 15 exhibited maximum cytotoxic activity against THP-1 cells. Moreover, Act 2 and Act 12 reduced cyclooxygenase (COX-2) and lipoxygenase (LOX) activity, which is involved in the proliferation and differentiation of cancer cells and may represent a possible molecular mechanism underlying leukemia growth inhibition. The bioactive antioxidant extracts of the selected *Streptomyces* species inhibited leukemia cell growth by reducing the COX-2 and LOX activity. Overall, our study not only introduced a promising natural alternative source for anticancer agents, but it also sheds light on the mechanism underlying the anticancer activity of isolated actinomycetes.

## 1. Introduction

Although great attention has been paid to developing anti-disease drugs, many of these diseases remain uncontrolled. Thus, it is of interest to search for new anti-disease agents, such as anti-cancer and anti-inflammation agents, that have different modes of action and sites of activity with no side effects [1]. Cancer can be considered as one of the most life-threatening diseases that influence the life of human beings. So far, several types of cancer are involved in the high mortality rates of roughly millions of patients every year. Leukemia represents the most common type of childhood cancer, diagnosed in children younger than 15 years [2]. Leukemia is a hemopoietic cancer that encompasses various biological distinctive subcategories [3]. Each year approximately 0.35 million cases of leukemia are diagnosed and the mortality rate in adults and children due to leukemia is 74% [4]. On the other hand, inflammation is usually triggered by damage to living tissues resulting from bacterial, fungal, and viral infections, and defective immune responses. It has also been demonstrated that there is a strong and complex interconnection between oxidative stress and the inflammatory response [5]. Many nonsteroidal anti-inflammatory drugs can reduce pain and inflammation by blocking the activity of the cyclooxygenase enzyme (COX) [6]. Unfortunately, there are many side effects associated with the administration of nonsteroidal anti-inflammatory drugs.

In this regard, scientists have recently focused their attention on the use of novel natural products or their derivatives from medicinal plants and microorganisms as alternatives to reduce the high risks and limits of chemotherapy [7]. Microbial diversity includes an infinite source of novel chemical compounds, offering valuable biotic agents for innovative biotechnology. Over recent years, much attention has been paid towards microorganisms, in particular bacteria that represent a significantly promising resource for antibiotics and other innovative bioactive natural products [8]. For example, actinomycetes, the filamentous Gram-positive bacteria that belong to the phylum Actinobacteria, constitute about 70% of the known bioactive metabolites such as antibiotics, immunosuppressive agents, antitumor agents, antioxidants and other therapeutic compounds [9]. To begin with, these antioxidant compounds are capable of scavenging the free radicals that induce oxidative stress and tissue damage. However, the microbial metabolites represent a promising tool for cancer prevention although the etiology is still obscure. Many experimental in vitro studies have been carried out using actinomycetes for the treatment of different cancerous cell lines. Active compounds or secondary metabolites produced by actinomycetes have the ability to control different tumor cells. Actinomycetes were found to have several compounds with anti-tumor activity such as anthracyclines, indolocarbazoles, macrolides, enediynes, isoprenoides, non-ribosomal peptides and others [10,11]. 

There are many diverse mechanisms by which these compounds could affect leukemias, such as inhibiting or blockage of signal transduction pathways inhibiting key enzymes, e.g., cyclooxygenase-2 activity, a rate-limiting enzyme for the synthesis of prostaglandins that are involved in the proliferation and differentiation of leukemia cells and ROS production, and lipoxygenase, which metabolizes arachidonic acid to hydroxyl eicosatetraenoic acids and leucotrienes, which suppress apoptosis and stimulate cell division of tumor. Recent studies suggest the use of natural antioxidants as adjuvant for the immune system in combination with chemotherapy [12]. The use of natural antioxidants during chemotherapy has been used for different cancer treatments because of their effective benefits that boost the immune response. Several trials have been recently established for the utilization of microbial extract, as a rich source of novel antioxidants [13], in an attempt to change the traditional chemotherapy treatment of cancer to biological treatment. This recent trial was planned to treat different types of cancer and, in particular, leukemia by using actinomycetes or their novel active compounds. These novel compounds from actinomycetes have evolved to influence very specific targets. Therefore, it could offer a significant means of changing cancer treatment from chemotherapy to biotherapy, using biological agents with minimal or no adverse effects and this is the major aim of the current study. Otherwise, this technology is still under investigation. 

Moreover, scarce data are available about the use of soil actinomycetes extracts in cancer treatment [14,15]. Few studies have investigated the potential use of actinomycetes extracts to treat inflammation and leukemia diseases [12]. Therefore, in the present study we will focus on selecting bioactive actinomycetes isolates and screening their extract impact on various leukemia cells, i.e., HL-60, K562 and THP-1. Moreover, to elucidate the possible molecular mechanisms by which actinomycetes inhibit leukemia growth, we will study COX-2 activity, a rate-limiting enzyme for the synthesis of prostaglandins that are involved in the proliferation and differentiation of several leukemia cells as well as the lipoxygenase activity, which is involved in stimulating cell division of tumor and ROS production in leukemia cells.

## 2. Materials and Methods

### 2.1. Isolation of Actinomycetes

**Figure 1:**
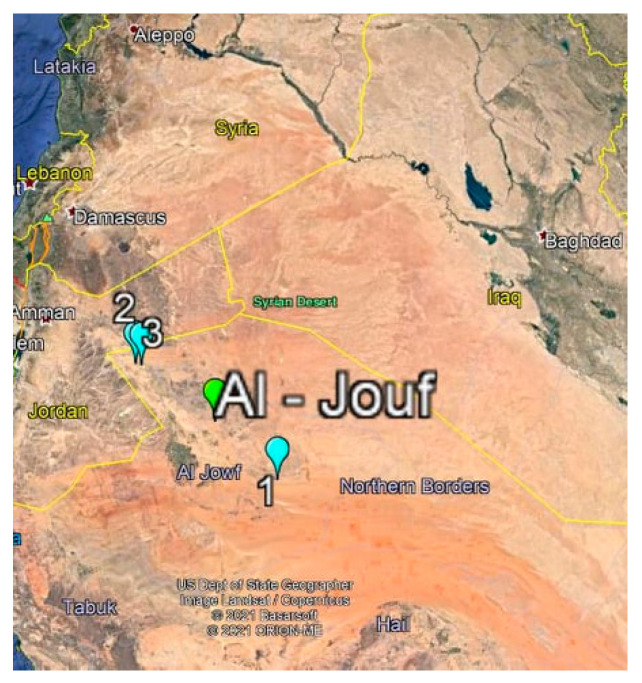
Map of Saudi Arabia showing the three soil sampling sites (Dumat Al-Jandal; Qasr Kaff and Ain Hawas Regions) of the Al-Jouf Region area.

The soil samples containing actinomycetes were collected from different barely explored sites in Al-Jouf province desert of Saudi Arabia (29°23.465′ N, 39°40.397′ E). Soil samples were taken from the surface of three different sites (Dumat Al-Jandal; Qasr Kaff and Ain Hawas Regions) (Figure 1). Al-Jouf province is located in the north of the country, bordering Jordan, and covers about 100,212 km² of area. The actinomycetes were isolated following the protocol described by [16], using glycerol-yeast agar medium supplemented with nystatin (50 μgL^−1^). One gram of soil was shaken with 10 sterile saline solution and then heated at 50 °C for 30 min. Serial dilutions were applied with a pour-plate method, followed by incubating the plates at 27 °C for 2 weeks. Purification of the selected colonies was done on glycerol-yeast agar medium at 27 °C for one week, and then the purified colonies were kept on starch casein agar as agar-slants at 4 °C, and as suspensions at −20 °C in (20%) glycerol [17]. 

### 2.2. Extraction of DNA from the Most Potent Actinobacterial Isolates

In order to extract genomic DNA, pure colonies of the five selected actinobacterial isolates were grown on the yeast extract malt extract dextrose medium for 2 days and the cells were precipitated by centrifugation at 10,000 rpm for 5 min, washed once with 500 µL phosphate-buffered saline (PBS) at pH 7.4, and then the Genomic DNA extraction Kit (QIAGEN, Hilden, Germany) was used according to the instructions. The quality of DNA was evaluated by spectrophotometer (Jenway 6305; Staffordshire, UK) and agarose gel electrophoresis.

### 2.3. Polymerase Chain Reaction (PCR) Amplifications of 16S rDNA 

To identify the isolates using molecular biology techniques, PCR was performed to amplify the 16S rDNA of the most effective actinobacterial isolates using the universal PCR primers; forward primer 27 F (5′-AGAGTTTGATCMTGGCTCAG-3′) and reverse primer 1492 R (5′-TACGGYTACCTTGTTACGACTT-3′). PCR was prepared by mixing 25 μL DreamTaq master mix (2X), 200 nM from each primer (1 µL), 50 ng from actinobacterial genomic DNA as template (2 µL), and finally sterile distilled water up to 50 μL. The program of PCR was performed on GeneAmp 9700 thermal cycler (Applied Biosystems, USA) and programmed to initial denaturation at 95 °C for 4 min; denaturation at 95 °C for 0.5 min, annealing at 58 °C for 0.5 min, extension at 72 °C for 1.5 min for 25 cycles, and a final extension step at 72 °C for 10 min.

### 2.4. Sequencing and Phylogenetic Analysis

The actinobacterial amplicons were purified using PCR Purification kit (Biobasic, Canada) and sequenced directly using the same primers by the Macrogen company (Seoul, Korea) using Big-dye terminator chemistry using the standard manufacturer’s protocol. The assembled contig sequence from each isolate was generated from the forward and reverse sequence reads using DNAStar Lasergene software (V. 7). The National Center for Biotechnology Information (NCBI) BLAST server was used to compare the obtained contig sequence of actinobacteria to references of 16S rDNA gene sequences of other actinobacterial isolates in GenBank, and then the sequences were aligned automatically using MUSCLE [18,19]. The alignment was carefully checked and sequence positions that contained gaps were eliminated. The MEGAX software was used to construct the phylogenetic relationship with the five actinobacterial isolates by maximum likelihood method and Kimura 2-paramater model and evaluation was based on 1000 bootstrap replications.

The molecular identified actinomycetes strains were deposited in Microbial Culture Collection, College of Applied Medical Sciences, Jouf University, KSA under code numbers from JU219 to JU223.

### 2.5. Biological Activity

Culture media was harvested by centrifuging at 5000 rpm and 4 °C for 20 min and the collected supernatant was extracted in equal volume of ethyl acetate solvent with the separating funnel method. The precipitate cell biomasses were also extracted with acetylacetone and both extracts were evaporated under vacuum to obtain the crude. The dried crude extract was dissolved in ethanol (90%), and the stock concentration was prepared (50 mg/mL). According to our preliminary experiment, 5 mg of the crude extraction was selected to investigate the biological activity of selected actinomycetes, because it showed the highest antioxidant activity. Ethanol (90%) was used as a control and the bioactivity of extracts was noted based on the zone of inhibition.

### 2.6. Preparation of Actinomyces (ACT) Extract

The identified actinomyces species were cultivated on starch nitrate agar plate medium at 28 °C for 7–14 days (until complete sporulation). One-liter Erlenmeyer flasks, each containing 250 mL of ISP2 medium consisting of 4 g/L glucose, 4 g/L yeast extract, and 10 g/L malt extract, were inoculated with sporesuspension from well grown slants (only one slant was used to inoculate two flasks). The flasks were incubated at 30 °C using a rotary shaker (150 rpm) for 15 days. The cells were separated by centrifugation at 5000 rpm and 4 °C and both the cell-free supernatant and the cell biomasses were subject to extraction. The supernatant was extracted with ethyl acetate 3 times. However, the cell biomasses were extracted with acetone and then the acetone was evaporated under vacuum and the remaining water residue was re-extracted three times with acetylacetate [20].

### 2.7. Metabolites Determination

The total phenolic and flavonoid content of actinobacterial extracts was determined according to Folin–Ciocalteu, aluminium chloride colorimetric and Nelson’s assays, respectively [21,22], while the individual phenolic acids and flavonoids were identified by homogenizing 50 mg of samples in a solution of acetone–water (4:1 *v*/*v*) for 24 h. HPLC (SCL-10A vp, Shimadzu Corporation, Kyoto, Japan), equipped with a Lichrosorb Si-60, 7 μm, 3 × 150 mm column, diode array detector), was used for quantification of phenolic compounds, whereas water:formic acid (90:10 (*v*/*v*)), and acetonitrile:water:formic acid (85:10:5 (*v*/*v*/*v*)) were applied as a mobile phase at a flow rate of 0.8 mL/min. Meanwhile, 3,5-dichloro-4-hydroxybenzoic was used as an internal standard. Finally, the concentration of each compound was detected by using a calibration curve of the corresponding standard, as previously described by [23]. Determination of tocopherols was done following the protocol described by [24], where tocopherols were extracted in hexane and quantified by HPLC (Shimadzu) by using dimethyl tocol (DMT) as internal standard (5 ppm). For quantification of carotenoids, a C18-column (Waters Spherisorb, 5 μm ODS1, 4.6 × 250 mm, with solvent A 81:9:10 acetonitrile:methanol:water and solvent B 68:32 methanol:ethyl acetate) was used, where the concentrations were determined using the Shimadzu Lab Solutions Lite software and a calibration curve [25].

### 2.8. Biological Activity

The total antioxidant capacities of actinobacterial extracts were evaluated using different assays, i.e., ferric reducing antioxidant power (FRAP), diphenylpicrylhydrazyl (DPPH) and 2,2′ azino bis (3-ethylbenzothiazoline-6-sulfonic acid) (ABTS), Superoxide scavenging capacity (SOS), xanthine/xanthine oxidase (X/XO) inhibition, anti-hemolytic, anti-lipid peroxidation and inhibition of hemolysis, following the protocols previously mentioned by [23,26,27,28]. The DPPH assay was done by taking a certain volume of each plant extract and mixing with an equal volume of DPPH solution (0.25 mM in 95% ethanol), then the mixture was left at room temperature for 30 min. The absorbance was measured at 517 nm and the inhibition percentage was calculated. For FRAP assay, 20 μL of each ethanol extract was mixed with FRAP reagent in a micro-titration plate. Then, it was incubated at 37 °C for 30 min, and then the absorbance was read at 593 nm. A Trolox calibration curve was used for calculating the antioxidant capacity of the extracts. The ABTS radical was prepared by mixing ABTS with 2.4 mM potassium persulphate, which were allowed to react for 12 h in the dark at room temperature and the absorbance was measured at 734 nm.

Based on their ability to oxidize nitrobluetetrazolium (NBT, yellow) to formazane (blue), superoxide radicals formed by the xanthine/xanthine oxidase (X/XO) system were evaluated by using 3 mL of 60 mM phosphate buffer, pH 7.4, which contains 30 mM xanthine, 30 mM EDTA, and 3 mM NBT. The reaction starts with addition of 5 U of xanthine oxidase, resulting in a superoxide anion, and hence, formazane formation. The scavenging activity of the extracts was determined by their ability to inhibit the reaction [26] and was evaluated as a decrease of 560 nm in the presence of extracts in comparison to the control. Superoxide dismutase (SOD) was used as a reference.

Deoxyribose (DR) (2.8 mM) in 20 mM phosphate buffer at pH 7.4 was oxidized by hydroxyl radical formed by the reaction of 100 pM iron (111), 100 pM ascorbate and 1 mM hydrogen peroxide, in the presence or absence of the extracts at 37 °C for 1 h [29]. Then, the oxidation products were mixed with thiobarbituric acid (TBA) 0.25 mM in 7% percloric acid at l00 °C for 10 min, and the resulting products were measured at 532 nm after addition of ethanol:ether (3:1). In this case, the hydroxyl radical would be able to inhibit the oxidation of DR, as well as the formation of thiobarbituric acid reactive substance (TBARS). Mannitol was used as a reference for comparing its scavenger activity with that of the extracts.

The antibacterial activity of the tested isolates was determined against *Streptococcus* sp, *Staphylococcus aureus*, *Escherichia coli*, *Bacillus cereus*, *Enterococcus faecalis*, *Salmonella* Typhimurium and *Pseudomonas aeruginosa*, using 100 μL of suspension containing 10^8^ CFU/mL of bacteria spread on Muller Hinton agar. The presence of inhibition zones was measured by Vernier caliper using the disc diffusion method based on the bacterial protocol MO2-A12 from the Clinical and Laboratory Standards Institute as mentioned previously [30]. The experiments were performed in independent triplicates and the cell-free actinobacterial extract diameters of the inhibition zones were measured and expressed in millimeters.

For determination of cytotoxic activity, human umbilical vein endothelial cells were cultured in Dulbecco modified essential medium in 5% CO_2_ at 37 °C. Then, cell lines were trypsinized, and resuspended in culture medium, then plated in a 96-well microtitre plate and incubated for 24 h. Afterwards, the actinobacterial extracts dissolved in DMSO were added to the cells, then incubated at 37 °C in 5% CO_2_ for 24 h, while the cells treated with DMSO without extract represented the negative control. Finally, cell viability was determined by MTT reagent [31].

### 2.9. In Vitro Cancer Cell Viability Assays

The impact of 90% ethanol extracts of the tested isolates and three blood cells (HL-60, K562 and THP-1) were used in this study. The cells were cultured in modified RPMI-1640 complete medium with 2.05 mM L-glutamine and 25 mM HEPES, supplemented with 10% heat inactivated FBS at 37 °C in a humidified atmosphere with 5% CO_2_. Cell counts and viability estimation by trypan blue dye exclusion test were performed regularly. Throughout the study procedures, cancer cells were maintained in a logarithmic growth phase at a concentration between 10^6^–10^7^ cells/mL. Media feeding was performed periodically every 2–4 days.

The effects of PGPE on leukemic cells were determined by conventional trypan blue exclusion assay (TBEA). The cells were treated with different concentrations of 90% ethanol extracts (0, 10, 25, 50, 100, 200, 300, 400, 800 µg/mL) in triplicate incubated for 72 h. The cells were harvested and counted with the haemocytometer after the treatment. Cell viability was recorded as a percentage of surviving cells following PGPE treatment. The data represent means from three independent experiments. Inhibitory concentration to be reduced by half (IC_50_) was calculated from the graph. 

### 2.10. Determination of Lipoxygenase (LOX) and Cyclooxygenase (COX) Activities

The anti-lipoxygenase activity was determined by using linoleic acid as a substrate and LOX as an enzyme, where 10 μL (50 mg/mL) of the potent extracts were added to 90 μL of LOX (400 U/mL) and incubated in the dark for 5 min at 25 °C. Then, 100 μL of linoleic acid (0.4 mM) were added to each well, then the reaction was incubated again in the dark for 20 min at 25 °C. Afterwards, 100 μL of freshly prepared ferrous orange xylenol (FOX) reagent, containing 90% methanol, 10 μM FeSO_4_, 100 μM xylenol orange, and 30 mM H_2_SO_4_, were added, and the reaction was kept for 30 min at 25 °C. Finally, the reading was measured at 560 nm and percentage of inhibition was calculated [32]. Meanwhile, cyclooxygenase-2 activity was evaluated following the manufacturer’s instructions of the COX assay kit (Cayman chemical company, Ann Arbor, MI, USA), where the microtitre plate was covered with a plastic film and kept at room temperature on a shaker for 18 h, then incubated in the dark for 90 min at 25 °C, and eventually the absorbance was measured at 420 nm and the percentage of inhibition was calculated [32].

### 2.11. Statistical Analyses

All of the experiments were statistically analyzed using the SPSS statistical program (SPSS Inc., Chicago, IL, USA). The One-way Analysis of Variance (ANOVA) was applied to all experimental data. Each experiment was replicated at least three times (n = 3–5). Hierarchical clustering (Pearson correlation) was generated by the Multi-Experimental Viewer (TM4 software package).

## 3. Results

### 3.1. Morphological and Chemical Characterization of the Isolated Actinomycetes

A total number of 21 different actinomycetes have been isolated and characterized in terms of morphological, biochemical and physiological attributes (Table 1). Morphological characterization of the isolates has shown that most of them belong to genus *Streptomyces* with extensively branched mycelia and coiled spore chains. Variations in the substrate color and aerial mycelia were observed for all the tested isolates, in addition to their ability to produce diffusible pigments (Table 1). Aerial hyphae of all isolates possessed mostly long spiral spore chains, long rectiflexible spore chains, or verticillate spores (Table 1). Moreover, all isolates showed biochemical differences regarding their ability to utilize various carbon sources (e.g., glucose, sucrose, galactose) and nitrogen sources (e.g., cysteine, valine, asparagine) for their growth, and also their ability to produce antioxidant enzymes such as catalase, peroxidase and asparaginase (Table 1).

### 3.2. Selection of the Most Biologically Active Actinomycetes 

**Figure 2:**
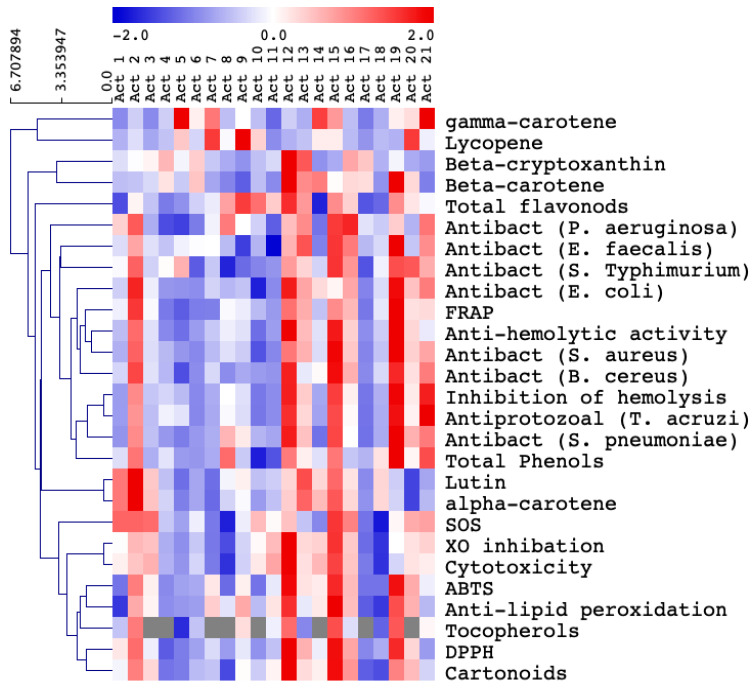
Hierarchical clustering (Pearson correlation) was generated to study bioactivity of several bacterial isolates. The measured parameters are represented by antioxidant metabolites and enzymes, overall antioxidant capacity (FRAP, DPPH, ABTS, SOS and XO inhibition), anti-hemolytic and anti-lipid peroxidation as well as their antibacterial and antiprotozoal activity. Data are represented by the means of at least three replicates.

Based on their higher levels of bioactive compounds as well as their prominent antioxidant, antibacterial and antiprotozoal activities, five isolates (i.e., Act 2, Act 12, Act 15, Act 19 and Act 21) were selected as the most potent ones (Table 2, Table 3 and Table 4, Figure 2). Obviously, isolate 12 appeared to have the highest content of the tested bioactive metabolites (flavonoids, phenolics, tocopherols and carotenoids), which were reflected by its relatively higher levels of antioxidant, cytotoxic, bactericidal and antiprotozoal effects. Isolate 15 was the second richest one in measured bioactive metabolites content and biological activity, followed by isolate 19 (Table 2, Table 3 and Table 4, Figure 2).

### 3.3. Molecular Identification and Phylogenetic Analysis of the Active Actinobacteria

**Figure 3:**
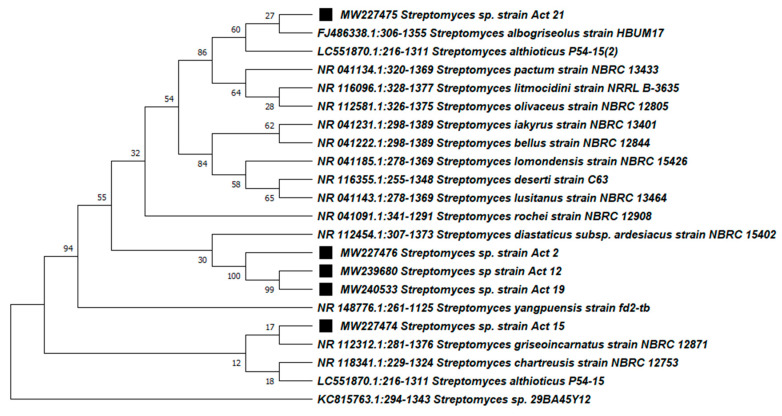
The evolutionary analysis of the five most biologically active actinobacterial isolates as analyzed by phylogenetic tree constructed by the maximum likelihood method using MEGAX software for the 16S rRNA sequences of *Streptomyces* spp. Act 2, Act 12, Act 15, Act 19 and Act 21. The numbers at nodes represent the percentage values given by 1000 bootstrap samples analysis.

The best biologically active actinobacterial isolates, namely Act 2, Act 12, Act 15, Act 19 and Act 21, were further subjected to molecular analysis to confirm the identity by sequencing the 16S rDNA gene using the universal primers 27 and 1492R (Figure 3). All isolate amplicons exhibited a high degree of sequence similarities with 16S rDNA from other *Streptomyces* species in GenBank ranging from 99.63–100%. The results revealed that all of the isolates belonged to genus *Streptomyces*, which confirmed the previous morphological and biochemical characterization (Figure 3). The isolate Act 2 showed high sequence homology similarities to many species of Streptomyces such as *Streptomyces yangpuensis, Streptomyces manipurensis* and *Streptomyces amritsarensis,* isolate Act 12 with *Streptomyces rochei* and *Streptomyces mutabilis*, isolate Act 15 with *Streptomyces chartreusis* and *Streptomyces variabilis,* isolates Act 19 with *Streptomyces longispororuber* and *Streptomyces thermocarboxydus* and isolate Act 21 with *Streptomyces litmocidini* and *Streptomyces olivaceus* (Figure 3).

The five actinobacterial isolate sequences were deposited into the GenBank database under the accession numbers MW227476, MW239680, MW227474, MW240533 and MW227475 for the isolates Act 2, Act 12, Act 15, Act 19 and Act 21 respectively. The MEGAX software was used to build the phylogenetic relationship with the related *Streptomyces* species using the maximum likelihood method and was evaluated by bootstrap analyses based on 1000 analysis (Figure 3). The phylogenetic tree separated the selected actinobacterial isolates into three different main clusters, *Streptomyces* sp. Act 15 in one main cluster, Act 21 in one cluster, and Act 2, Act 12 and Act 19 in the same cluster (Figure 3).

### 3.4. Phenolic Profile of Selected Actinomycetes Isolates 

From the present results, gallic acid was observed to be the most dominant phenolic compound in all the selected isolates (Act 2, Act 12, Act 15, Act 19 and Act 21) (Table 5). Meanwhile, isolate 21 showed a great diversity in phenolic content among the selected isolates, containing the highest amounts of ferulic, protocatechuic, galic, p-coumaric, chlorogenic, sinapic and ellagic acids, and catechin, resorcinol, quercetrin, isoquercetrin, rutin, velutin, naringenin, genistein, fisetin and O-hydroxydaidzein, while isolate 19 had the highest levels of caffeic acid, apigenin and daidzein (Table 5). The results obtained also indicated that isolate 2 and 12 contained the highest values of quercetin and luteolin, respectively. Some phenolic compounds were not detected in some isolates, e.g., apigenin was absent in isolate 2, 15 and 21; and velutin was not detected in isolate 2, 15 and 19 (Table 5).

### 3.5. Biological Activities of Selected Actinomycetes Isolates towards Inflammation and Leukemia

**Figure 4:**
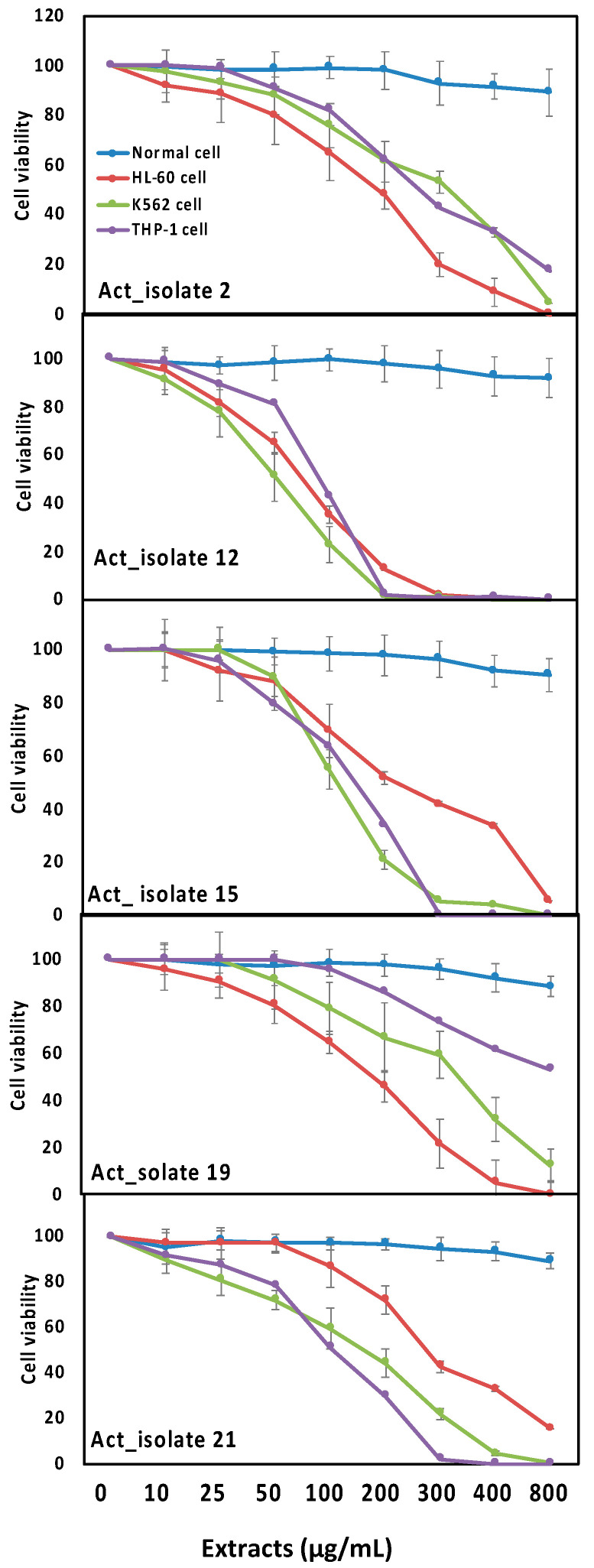
The activity of 90% ethanol extracts (0, 10, 25, 50, 100, 200, 300, 400, 800 µg/mL) of five most biologically active *Streptomyces* isolates (Act 2, Act 12, Act 15, Act 19 and Act 21) against leukemia cell viability (HL-60, K562 and THP-1). Data are represented by the means of at least 3 replicates and error bars represent standard deviations.

**Figure 5:**
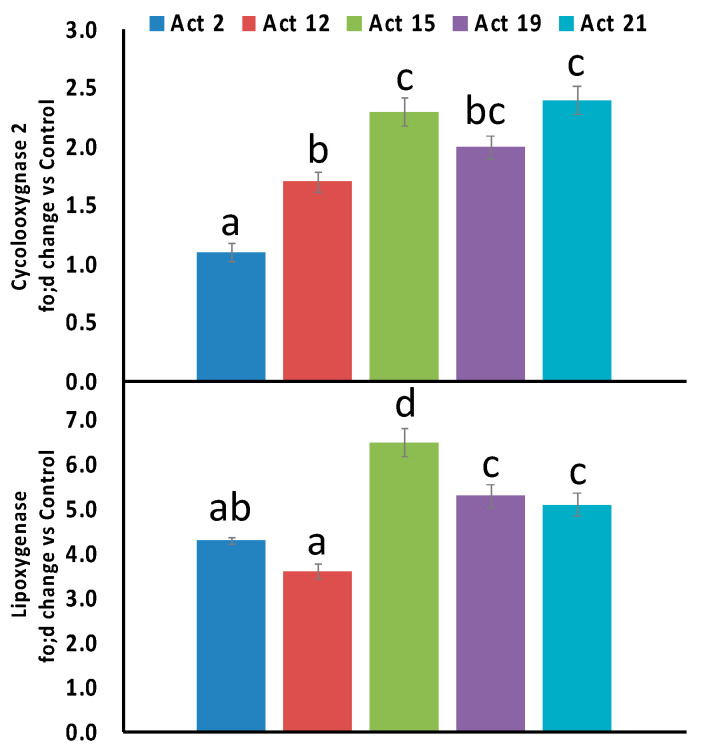
The effect of the 90% ethanol extract of the five most biologically active *Streptomyces* isolates (Act 2, Act 12, Act 15, Act 19 and Act 21) on cyclooxygenase (COX-2) and lipoxygenase (LOX) activity. Data are represented by the means of at least 3 replicates and error bars represent standard deviations. Different letters above the bars indicate significant differences (*p* < 0.05, Tuckey test).

According to the current results, the tested cell lines (HL-60, K562 and THP-1) experienced different concentrations of isolates 2, 12, 15, 19 and 21 (Figure 4). In general, the five active isolates showed cytotoxic activity against the tested cell lines in a dose dependent manner. Among the potent isolates, isolate 12 significantly decreased the cell viability and showed maximum cytotoxic activities against both HL-60 and K562 cells with IC50 values of 142.42 and 48.89 respectively, while isolate 15 exhibited maximum cytotoxic activity against THP-1 cells with an IC50 of 78.39 (Figure 4). Regarding the anti-inflammatory properties, the results shown in Figure 5 revealed that, among the five tested isolates, isolate 2 and 12 caused significant reductions in COX-2 and LOX inflammatory markers, indicating that both isolates had the highest anti-inflammatory activities.

## 4. Discussion

### 4.1. Characterization of the Isolates

Morphological identification of the tested actinomycetes isolates has revealed that most of them belong to the genus Streptomyces with extensively branched mycelia and coiled spore chains (Table 1) [33,34]. In addition, the development of aerial hyphae into spores is frequently one of the key features for identification of Streptomyces species [35]. It has been also previously demonstrated that the majority of soil isolates have aerial coiled mycelia with spore chains [36]. Moreover, the availability of carbon and nitrogen sources, utilized by the tested isolates, might have a great influence on types and amounts of bioactive secondary metabolites synthesized by Streptomyces in response to composition of substrates (Table 1 and Table 4, Figure 2) [37]. Furthermore, the production of enzymes by actinomycetes could be regarded as an indication for their abilities to survive in competing environments (Table 1) [38]. According to our results in Figure 3, Streptomyces such as *Streptomyces albogriseolus* and *Streptomyces yangpuensi*, which have high sequence homology similarities to our isolated strains, showed a wide range of biological activities including antibacterial [39,40].

### 4.2. Biological Activities and Secondary Metabolites of Actinomycetes Contribute to Determination of the Most Active Isolates

Actinomycetes, particularly the genus *Streptomyces*, are known as a potent source of secondary metabolites that have a wide array of biological activities [41,42]. In the current study, the extracts of all isolates were evaluated for their content of biologically active metabolites such as flavonoids, phenolics, tocopherols and carotenoids (Table 4, Figure 2). Moreover, the antioxidant properties measured using different assays (FRAP, DPPH, ABTS, SOS, XO inhibition, anti-hemolytic, anti-lipid peroxidation and inhibition of hemolysis) as well as the cytotoxic, antibacterial (*Streptococcus* sp, *Staphylococcus aureus*, *Escherichia coli*, *Bacillus cereus*, *Enterococcus faecalis*, *Salmonella Typhimurium* and *Pseudomonas aeruginosa*) and antiprotozoal activities (*Trypanosom acruzi*) of all isolates were also determined (Table 2 and Table 3, Figure 2). In this regard, the *Streptomyces* genus has an excellent track record for the discovery of secondary metabolites [42]. The findings from our study (Table 2, Figure 2) are also in agreement with previous reports of [43], who stated that *Streptomyces* species isolated from medicinal plants have antimicrobial and antitumor properties. Furthermore, antioxidant, antimicrobial, and hypertension activities have been reported for similar isolates [44,45]. The presence of high antioxidant as well as activity against tested bacterial pathogens guaranteed further study to find potential antimicrobial compounds (Table 3 and Table 4).

Interestingly, a positive correlation was found between the bioactive metabolite contents and biological activities of the five selected isolates, which is in line with previous studies by [13], who observed a strong correlation between free radical scavenging activity and phenolic compound content of actinomycete isolates from the mangrove soils. Other studies have also shown that actinomycetes are a rich source of antioxidant compounds [9]. Therefore, the biological activities of actinomycetes exhibited in this study might be attributed to the presence of appreciable amounts of phenolics and flavonoids (Table 4, Figure 2) [46,47]. The presence of higher amounts of carotenoids might also take part in the total antioxidant capacity of actinomycete isolates (Table 4, Figure 2). For instance, *Streptomyces griseus* subsp. *griseus* has been previously found to produce isorenierantene, an aromatic carotenoid [48], which was shown to have antioxidant activity [49]. Thus, the remarkable antioxidative properties of such isolates might recommend them as potential contributors to treatment of various diseases caused by generation of reactive oxygen species.

In agreement with our findings (Table 4, Figure 2), it has been previously reported that the higher levels of phenolic compounds of actinomycetes could be associated with their enhanced biological activities [46,47]. Similar Streptomyces species such as *Streptomyces lavendulae* have been shown to exhibit high levels of antioxidant activities [46]. In general, microbial extracts have been recently investigated for their utilization as a rich source of novel antioxidants [13]. The antibacterial activities displayed by some actinomycetes isolates against *Klebsiella* sp., *E. coli*, *Proteus* sp., *Staphylococcus* sp., *Salmonella* sp. and *Bacillus* sp. are also in line with previous studies by [50].

Our findings could also be compared with various studies, whereas different strains of *Streptomyces* sp. have been previously shown to possess significant DPPH and ABTS antioxidant activities [51]. For instance, *Streptomyces lavendulae* exhibited promising antioxidant potential by DPPH, lipid peroxidation and hydroxyl radical scavenging activities [46]. Moreover, previous studies have demonstrated that several strains of actinomycetes exhibited strong antibacterial potency against some pathogenic bacteria such as *Bacillus subtilis, Bacillus cereus, Staphylococcus aureus, Escherichia coli, Proteus vulgaris, Pseudomonas aeruginosa*, *E. amylovora*, *A. tumefaciens*, *Michiganensis* sp., *P. viridiflova*, *C. michiganensis*, *E. feacalis*, *K. pneumoniae*, *S. lutea*, *S. epidermidis*, *Xanthomonas* sp. and *Pseudomonas vulgaris* [52,53,54,55,56]. Moreover, actinomycetes isolated from mangrove samples have induced antibacterial activity against *Klebsiella* sp., *E. coli*, *Proteus* sp., *Staphylococcus* sp., *Salmonella* sp. and *Bacillus* sp. [50]. The antiprotozoal activity of some *Streptomyces* isolates against the malarial parasite *Plasmodium falciparum* has also been well documented [57]. 

The phenolic profile of the selected potent isolates was analyzed in order to highlight the major compounds that might be responsible for their bioactivities (Table 5), whereas the five active isolates showed high variations in their content of phenolic compounds. In agreement with our results (Table 5), some phenolic derivatives with high antioxidant potential were previously isolated from a *Streptomyces* sp. [58]. Other studies have also demonstrated that actinomycetes have several compounds with anti-tumor activity such as anthracyclines, indolocarbazoles, macrolides, enediynes, isoprenoides, non-ribosomal peptides and others [10,11].

Likewise, several phenolic compounds have been previously isolated from actinomycetes strains. For example, about 12 flavonoid compounds have been isolated from *Actinomadura miaoliensis*; namely 5-hydroxy-4′,7,8-trimethoxyflavone, 5-hydroxy-3′,4′,7,8-tetramethoxyflavone, 5-hydroxy-4′,7-dimethoxyflavone, 1-O butyl-b-d-fructopyranoside, stigmastane-3b,5a,6b-triol, (2Z)-3-{4-[(3-methylbut-2-en-1-yl)oxy]phenyl}prop-2-en-1-ol, (E)-ferulic acid hexacosyl ester, tricosanoic acid, 6b-hydroxystigmast-4-en-3-one and 6b-hydroxystigmasta-4,22-dien-3-one, balansenates I and II, and 9-(3-methylbut-2-en-1-yl)adenine [59]. Further, two phenolic compounds; namely phenol,2,5-bis(1,1-dimethylethyl)- and phenol, 2,20-methylenebis[6-(1,1-dimethylethyl)-4-methyl-], were identified from a *Streptomyces* sp. isolated from mangrove soil, and have proved to be incorporated in the antioxidant activity (DPPH, ABTS and lipid peroxidation) exhibited by such actinomycete species [58]. These previous findings could extensively support our obtained results and appreciate the contribution of phenolic compounds to the biological activities of actinomycetes, particularly the antioxidant and antibacterial activities.

### 4.3. Production of Phenolic Compounds and Asparaginase by Actinomycetes Supports Their Anti-Leukemic Activities

Based on their richness in bioactive compounds, actinomycetes have been considered as antitumor agents [10,11]. The present study has also explored the potential effects of the biologically active actinomycetes on targeting leukemia (Figure 4), where the five active isolates showed cytotoxic activity against the tested cell lines (HL-60, K562 and THP-1).

In line with our results (Figure 4), previous studies have shown that some *Streptomyces* species are able to exert antitumor activity against leukemia [14,60]. Moreover, some other *Streptomyces* species have been shown to have anticancer activity against different types of cancer cell lines, i.e., A549, HeLa, PC-3, THP and Caco-2 [46,61]. Similar results have also been previously obtained for *Streptomyces griseus*, which induced anticancer activities against hepatocellular and breast cancer cells [62].

Similar to our results, it has been revealed that *Streptomyces* sp. SF2575 [60] and *Actinomadura* sp. [63] showed antitumor potency against P388 lymphotic leukemia. In addition, *Streptomyces avermitilis* reportedly induced inhibitory activities against chronic myelogenous leukemia and colon carcinoma cell lines [14], whereas *Streptosporangium* sp. exhibited cytotoxic activity towards IPC-81 rat leukemia cells [64]. In comparison with our results, numerous species and strains of actinomycetes have been reported to produce inhibitory effects against different cancer cell lines. For example, *Streptomyces collinus* showed marked cytotoxic efficacy against HeLa, PC-3, THP and Caco-2 cancer cell lines [61], *Streptomyces lavendulae* caused cytotoxic effects against the A549 lung adenocarcinoma cell line [46], and *Nocardia mediterranei* subsp. *kanglensis* displayed cytotoxicity against colorectal cancer cells [65]. Further, *Streptomyces griseus* exhibited antitumor activity against hepatocellular and breast carcinoma cells [62], and *Streptomyces scabrisporus* showed antiproliferative activity against N2a, MCF-7, MiaPaca-2, PC-3, HCT-116, MDA-MB-231, HL-60 and A-549 cell lines [66].

In the same context, some of the flavonoid compounds described herein (Table 5) could be compared with those previously reported, regarding their role in cytotoxic activities and induction of apoptotic machinery, in relation to their mechanisms of action. For instance, quercetin caused apoptosis in different cancerous cell lines [67], and inhibited the formation of Bcl-2 proteins in different tumor cells such as the acute leukemia cell line HL-60 [68,69]. Rutin was also supposed to act by induction of apoptosis and cell cycle arrest in several cancer cell lines [70,71], in addition to its antiviral, anti-inflammatory, antitumor and antioxidant activities [72,73]. Similarly, the anti-leukemic activities of other flavonoids such as baicalein, luteolin, genistein, apigenin, scutellarin, galangin, chrysin, and naringenin against various kinds of leukemia cells (HL-60, NB4, U937, K562, Jurkat) have been previously investigated [74]. Moreover, a flavonoid compound (quercetin-3-O-β-L-rhamnopyranosyl-(1→6)-β-D- glucopyranoside) was previously isolated from *Streptomyces* sp. and induced cytotoxic potential against A549 lung adenocarcinoma cell lines by inducing apoptosis through enhancing the activity of caspase-3 and caspase-9 and inhibiting the synthesis of Bcl-2 protein as well as cytochrome c release from mitochondria [15]. A recent study by [75] has shown that metabolites extracted from *Streptomyces levis* induced apoptosis via increasing the expression of caspase-3 and decreasing that of Bcl-2 in acute lymphoblastic leukemia. Thus, a similar mechanism could be proposed for the inducible anti-leukemic activities of the biologically active isolates (2, 12, 15, 19, and 21), which might illustrate the crucial role of the quantified phenolic compounds in targeting different types of leukemia.

On the other hand, actinomycetes are not only a large store of diverse secondary metabolites, but they are also powerful producers of many biologically important enzymes [76]. The current investigation has shed light on the anti-leukemic enzyme L-asparaginase, which was produced by most of the studied isolates, especially the most biologically active ones (Act 2, 12, 19 and 21) (Table 1).

In agreement with our results (Table 1, Figure 4), L-asparaginase from *Streptomyces rochei* has been previously investigated for its anticancer activities against different kinds of cell lines (e.g., HeLa, HepG-2, MCF-7, Hep2 and Caco2) [77]. Moreover, previous studies have investigated the potential effects of L-asparaginase in treating different types of leukemia [78,79].

In this regard, previous reports indicated that actinomycetes have not been intensively studied compared to other L-asparaginase-producing organisms [80]. However, some species of *Streptomyces* such as *S. karnatakensis*, *S. venezualae*, *S. longisporusflavus*, *S. ginsengisoli* have been explored for their potentiality to produce L-asparaginase [81]. L-asparaginase has been largely involved in fighting acute lymphoblastic leukemia, principally by decomposing the higher levels of the amino acid asparagine, which is critically required by cancerous cells for their malignant growth, into aspartate and ammonia, thereby effectively killing the cancerous cells [82]. Pyrimidines, which form an essential part of the enzyme, might have a role in several biological properties such as anti-bacterial and anti-cancer activities, depending on the type of substituent attached to the pyrimidine ring [83]. Moreover, L-asparaginase has been recently shown to induce apoptosis in chronic myeloid leukemia cells [84]. Former studies have investigated the inhibitory activities of L-asparaginase, purified from *Streptomyces rochei* subsp. *chromatogenes*, against various kinds of cancer cells (e.g., HeLa, HepG-2, MCF-7, Hep2 and Caco2) [77]. Interestingly, L-asparaginase has been used, combined with other drugs, for treatment of lymphocytic leukemia, chronic lymphosarcoma, acute myelomonocytic leukemia and acute myelocytic leukemia [78,79].

### 4.4. Anti-Inflammatory Activities of Selected Actinomycetes Isolates

Several inflammatory pathways have been involved in tissue damage after injury, such as cyclooxygenase (COX-2) and lipoxygenase (LOX). COX-2 is associated with the inflammatory tissue as an inducible isoform [85]. Meanwhile, COX-2 products might cause some side effects such as gastrointestinal irritation [86]. LOX products have been linked to numerous skin inflammations [86]. There are many diverse mechanisms by which these compounds could affect leukemias such as inhibition or blockage of signal transduction pathways, inhibiting key enzymes, e.g., COX-2 activity, a rate-limiting enzyme for the synthesis of prostaglandins, which are involved in the proliferation and differentiation of leukemia cells and ROS production. Similarly, LOX metabolizes arachidonic acid to hydroxyl eicosatetraenoic acids and leukotrienes, which suppress apoptosis and stimulate tumor cell division. Therefore, here we studied the impact of actinobacterial extracts on COX-2 and LOX activities (Figure 5).

In this regard, actinomycetes could represent an alternative natural source for anti-inflammatory products. The anti-inflammatory properties of actinomycetes might be ascribed to their rich content of flavonoids such as quercetin (Table 4 and Table 5), which has previously been found to induce strong inhibitory effects on COX-2 and LOX enzymes [87].

In agreement with our results (Figure 5), it has been previously reported that some flavonoid derivatives isolated from other actinomycetes species could produce anti-inflammatory activities against lipopolysaccharide (LPS)-induced tumor necrosis factor (TNF-a) [59,88]. While, some marine actinomycetes are capable of producing other compounds such as cyclomarin A and C, which have been shown to have anti-inflammatory effects [89]. The anti-inflammatory properties of actinomycetes could also support their antileukemic activities, as previously evidenced by the role of actinomycetes metabolites in inhibiting COX-2 and LOX enzymes [12].

Similar studies have demonstrated that some flavonoid compounds isolated from *Actinomadura miaoliensis*, namely 5-hydroxy-4′,7,8-trimethoxyflavone, 5-hydroxy-3′,4′,7,8-tetramethoxyflavone, and 5-hydroxy-4′,7-dimethoxyflavone, exhibited inhibitory effects on LPS-induced TNF-a production [59]. Moreover, *Actinomadura spadix* has been shown to inhibit the production of NO, TNF-α, and IL-1β on LPS-induced macrophages cells [88]. Other studies have reported that cyclopeptides such as cyclomarin A and C produced by marine actinomycetes (e.g., *Salinispora arenicola*) were responsible for its anti-inflammatory action [89].

## 5. Conclusions

Based on the above results, it could be concluded that actinomycetes might be considered as valuable sources of bioactive natural compounds. The most promising actinomycetes could be selected on the basis of their higher content of phenolic compounds and tocopherol, which consequently are expected to increase their antioxidant, antibacterial and antiprotozoal activities. Furthermore, such active isolates proved to have the ability to inhibit the growth of different leukemia cells, which could be associated with their potential to reduce cyclooxygenase (COX-2) and lipoxygenase (LOX) activities. Thus, our study not only introduced a promising natural alternative source for anticancer agents, but it also sheds light on the mechanism underlying the anticancer activity of isolated actinomycetes. However, this is still the first step and is still far away from being used in treatments. Therefore, extraction, purification and production of phytochemicals from bacterial isolates represent a promising approach to develop different therapeutic applications, the subject of future work. Further, more in vivo studies such as apoptosis and mitochondrial membrane potential assays are needed in the future to provide a more detailed and mechanistic view on how the active extracts and isolated secondary metabolites work against cancer disease.

## Figures and Tables

**Table 1:** The morphological and biochemical characterization of 21 bacterial isolates. The signs + and − indicate presence or absence, respectively.

	Isolate	1	2	3	4	5	6	7	8	9	10	11	12	13	14	15	16	17	18	19	20	21
**Colony**	Aerial mycelium	+	+	+	+	−	+	+	+	+	−	+	−	+	+	−	+	+	−	+	+	+
	Pigmentation	+	+	+	+	+	+	+	+	+	+	+	+	+	+	+	+	+	+	+	+	+
**Spore chain**	Spiral	+	+	−	+	+	−	−	+	−	+	+	+	−	+	−	+	+	+	+	+	+
	Rectiflexibels	−	−	+	−	−	+	−	−	−	−	+	−	−	−	−	−	+	−	−	−	−
	Verticillate	−	−	−	−	−	−	+	−	+	−	+	−	+	−	+	−	+	−	−	−	−
**Spore color**	Yellow	+	+	−	−	−	−	−	−	−	−	−	−	+	−	+	−	−	+	−	−	−
	Orange	−	−	+	+	−	+	−	+	−	+	−	+	−	−	−	+	+	−	+	−	+
	Red	−	−	−	−	+	−	+	−	+	−	+	−	−	+	−	−	−	−	−	+	−
**N source utilization**	L-Cysteine	+	−	−	+	+	+	+	+	+	−	−	+	+	−	+	+	−	−	+	−	+
	L-Phenylalanine	−	+	+	−	−	+	+	−	+	−	+	−	+	+	+	−	+	+	−	+	−
	L-Histidine	−	+	−	+	+	−	+	+	−	−	+	−	+	−	−	−	+	+	−	+	−
	L-Lysine	+	+	+	−	−	+	+	−	+	−	−	+	+	+	−	+	+	+	+	+	+
	L-Asparagine	+	+	−	+	+	−	−	+	−	+	+	+	+	+	−	+	+	−	+	−	+
	L-Arginine	+	−	+	−	−	+	+	−	+	−	+	+	−	−	−	+	−	−	+	+	+
	L-proline	+	+	−	+	−	−	+	+	+	−	−	+	+	+	+	+	+	−	+	−	+
	L-Valine	−	+	−	−	+	+	+	−	−	−	+	−	+	−	−	−	+	+	−	−	−-
	Tyrosine	+	+	−	+	−	+	−	+	−	−	+	+	−	+	−	+	−	−	+	−	+
**C source utilization**	D-fructose	−	−	−	+	+	−	+	−	−	+	−	−	+	−	−	−	+	+	−	+	−
	D-glucose	+	+	−	+	−	−	−	+	+	−	−	+	−	+	+	+	+	+	+	+	+
	Sucrose	−	+	+	−	+	−	+	−	+	+	+	+	+	+	−	+	+	−	+	+	+
	Maltose	−	−	−	−	+	+	+	−	+	+	+	−	+	−	−	−	−	−	−	−	−
	Raffinose	+	+	+	+	+	+	+	+	+	−	+	+	+	+	−	+	+	+	+	+	+
	Lactose	−	−	−	+	−	−	−	+	+	−	+	−	−	−	−	−	+	+	−	−	−
	Galactose	+	+	−	+	+	−	+	+	−	−	+	+	−	−	−	+	+	−	+	+	+
	Meso-Inositol	+	−	−	−	+	+	+	−	+	+	+	+	−	−	+	+	−	−	+	+	+
	Celullose	−	−	−	+	−	−	−	+	+	+	+	−	+	+	+	−	+	+	−	+	−
	Xylose	+	−	−	−	+	−	−	−	+	+	−	+	+	+	+	+	+	−	−	−	+
	Dextran	+	−	+	−	−	−	+	−	+	−	+	+	−	+	−	+	−	+	+	+	+
**Enzymes activity**	Catalase	−	−	+	−	+	−	+	−	+	−	+	−	+	−	−	−	+	−	−	+	−
	Peroxidase	+	−	−	+	+	+	+	+	−	+	−	+	+	+	+	+	−	−	−	+	+
	Starch hydrolysis	+	+	+	−	−	−	−	−	+	+	−	+	−	−	−	+	−	+	+	−	+
	Gelatin liquefication	+	−	−	+	−	+	−	+	+	+	−	+	+	−	+	+	−	+	+	−	+
	Casein hydrolysis	−	−	+	−	+	+	+	−	−	−	−	−	−	−	+	−	−	−	−	+	−
	Lipolysis	+	+	+	+	−	+	+	+	+	+	+	+	+	−	−	+	−	+	+	−	+
	Citrate utilization	+	+	−	+	+	+	−	+	+	+	+	+	+	+	+	+	−	+	+	−	+
	H_2_S Production	−	+	−	−	+	+	+	−	+	−	+	−	−	+	+	−	+	+	−	+	−
	DNase	+	−	−	+	−	−	+	+	−	+	−	+	+	−	−	+	−	−	+	+	+
	Nitrate reduction	+	+	+	−	−	−	−	−	+	+	−	+	−	−	+	+	+	+	+	+	+
	Urease	+	+	−	+	+	−	+	−	+	+	+	+	−	+	+	+	+	+	+	+	+
	L-asparaginase	+	−	+	−	+	+	−	−	−	+	+	+	−	+	−	+	+	−	−	+	+
	L-glutaminase	+	−	−	+	+	+	+	+	−	+	−	+	+	+	+	+	+	−	+	+	+

**Table 2:** The antioxidant, anti-hemolytic, cytotoxicity and antiprotozoal activity of 21 actinobacterial isolates. Data are represented by the means of at least 3 replicates ± standard deviations (SD).

Isolates	FRAP	DPPH	ABTS	SOS	XO inh	Anti-Hemolytic	Anti-Peroxi	Anti-Hemolysis	Cytotoxicity	*T. acruzi*
Act 1	42.4 ± 2	68 ± 4.4	30.5 ± 1.9	83 ± 10.2	75.2 ± 9.5	34.6 ± 2.3	17.9 ± 1.2	25.7 ± 1.7	61.3 ± 9.2	4.1 ± 0.3
Act 2	67 ± 5.2	97 ± 8	91.1 ± 8.8	83 ± 8.3	95.3 ± 9.5	61.4 ± 5.1	92.2 ± 9.8	44.5 ± 3.7	71.9 ± 7.8	7.1 ± 0.6
Act 3	43 ± 1.5	58 ± 2.3	65.5 ± 2.7	81 ± 4.4	92.8 ± 5	38.9 ± 1.8	72.7 ± 3	29.3 ± 1.4	77.1 ± 4.2	4.6 ± 0.2
Act 4	28 ± 2.1	24 ± 5.7	35.1 ± 11	42 ± 11.3	52.9 ± 11	28 ± 0	39.3 ± 13	30.7 ± 4.7	37.6 ± 10.4	5.4 ± 1
Act 5	25.3 ± 1	31.8 ± 1	40.5 ± 1.5	35.6 ± 1	44.8 ± 1.6	25.2 ± 1.6	41.6 ± 1.3	28.4 ± 1.3	30.5 ± 0.8	5.1 ± 0.2
Act 6	28 ± 2.2	39 ± 3.1	41.9 ± 3.4	50 ± 4.4	60 ± 5.1	30.5 ± 2.4	43.9 ± 3.9	23.9 ± 1.8	46.4 ± 4.1	3.8 ± 0.3
Act 7	29.3 ± 1	53.2 ± 3	67.9 ± 5.4	28 ± 0.9	38.7 ± 1.6	35.9 ± 2.6	82.5 ± 7	27 ± 1.9	23.9 ± 0.6	4.3 ± 0.3
Act 8	43.9 ± 3	38 ± 2.1	28.7 ± 1.3	12.0.9	27.5 ± 2.2	40.8 ± 3.2	61.5 ± 4.1	34.3 ± 2.7	6.8 ± 0.5	5.5 ± 0.4
Act 9	41 ± 2.5	56 ± 4.7	65.3 ± 6	50 ± 4.4	63.8 ± 5.3	39.1 ± 2.7	92.5 ± 7.4	31.5 ± 2.1	45.1 ± 4.1	5.1 ± 0.3
Act 10	36 ± 2.5	36.4 ± 1	30.4 ± 0.8	66 ± 4.6	77.7 ± 5.2	24.9 ± 1.5	51.6 ± 2.1	22.5 ± 1.4	63.3 ± 4.4	3.6 ± 0.2
Act 11	32.2 ± 2	47.6 ± 4	56.6 ± 5.3	55 ± 3.4	94.3 ± 6.9	30.7 ± 2.4	76.6 ± 6.5	24.4 ± 1.8	80.1 ± 5.9	3.9 ± 0.3
Act 12	61.4 ± 5	124 ± 9	115.3 ± 9	65 ± 4.9	167 ± 13	74 ± 6.2	123 ± 9.1	53.2 ± 4.5	139.8 ± 11	8.5 ± 0.7
Act 13	54 ± 3.2	81 ± 3.4	68.5 ± 2.7	42 ± 2.5	86.5 ± 3.1	51.7 ± 2.4	63.3 ± 2.3	39 ± 1.9	65.6 ± 2.3	6.2 ± 0.3
Act 14	37 ± 2.4	57.9 ± 4	66.9 ± 5.4	30 ± 2.3	88.4 ± 7.6	39.1 ± 2.8	75.9 ± 6.5	26.5 ± 1.8	75.3 ± 6.7	4.2 ± 0.3
Act 15	58.2 ± 5	117 ± 9	109.4 ± 8	91 ± 7.8	129 ± 9.3	70.8 ± 5.8	137 ± 10.5	50.8 ± 4.2	103.6 ± 7.1	8.1 ± 0.7
Act 16	52 ± 3.3	84 ± 4	77.4 ± 3.9	77 ± 4.5	90.7 ± 5.1	49 ± 2.3	86.6 ± 3.9	35.9 ± 1.7	72 ± 4.2	5.7 ± 0.3
Act 17	28.2 ± 1	33 ± 1.6	30.4 ± 1.3	27 ± 1.6	36.2 ± 2.1	28.8 ± 1.8	27.1 ± 1.2	22.4 ± 1.4	23.7 ± 1.4	3.6 ± 0.2
Act 18	41 ± 3	42.5 ± 3	30.5 ± 2.4	10 ± 1.1	21.4 ± 1.7	36.5 ± 2.8	18.5 ± 1.5	27.3 ± 2.1	6.5 ± 1	4.4 ± 0.3
Act 19	84 ± 6.8	112 ± 10	113.7 ± 11	55 ± 6	75.9 ± 7.5	76.5 ± 6.3	115.2 ± 12	55.4 ± 4.6	46.7 ± 5.3	8.9 ± 0.7
Act 20	48 ± 1.8	72 ± 2.8	81.4 ± 3.3	70.4.4	84 ± 5.2	48.3 ± 2.2	90.3 ± 3.7	36.3 ± 1.7	64.4 ± 4.2	5.8 ± 0.3
Act 21	49 ± 4	38 ± 10	57.8 ± 21	71 ± 2.5	88 ± 26.2	40.2 ± 0	50.6 ± 17.6	53.2 ± 8.7	62.4 ± 23.4	9.3 ± 1.9

**Table 3:** Screening for antibacterial activities of actinobacterial isolate cell-free extracts. Data are represented by the means of at least 3 replicates ± standard deviations (SD).

Isolates	*S. pneumonia*	*S. aureus*	*E. coli*	*B. cereus*	*E. faecalis*	*S. typhimurium*	*P. aeruginosa*
Act 1	9.8 ± 0.6	11 ± 0.8	13 ± 0.9	14.4 ± 1	15.7 ± 1	17.5 ± 1.1	19.1 ± 1.2
Act 2	16.3 ± 1.3	21 ± 1.8	24 ± 2	27 ± 2.2	21 ± 2.9	25.2 ± 0.7	24.9 ± 1.6
Act 3	11.1 ± 0.6	13 ± 0.6	14 ± 0.6	15 ± 0.6	15 ± 0.6	15.6 ± 0.5	12.5 ± 0.6
Act 4	9.9 ± 0.8	12 ± 1.1	10 ± 1.5	12.6 ± 1	14 ± 2.8	18 ± 6.5	7.8 ± 2
Act 5	9.2 ± 0.6	10 ± 0.5	10 ± 0.5	7 ± 0.4	16 ± 0.9	21.5 ± 0.4	7.2 ± 0.4
Act 6	9.9 ± 0.7	10 ± 0.8	10 ± 0.8	10 ± 0.9	17 ± 2.5	10.1 ± 0.9	10.1 ± 0.9
Act 7	10.5 ± 0.7	12 ± 0.9	13 ± 0.9	14 ± 1.0	17 ± 2.4	15.1 ± 1.3	16 ± 1.5
Act 8	15.2 ± 1.2	13.6 ± 1	12.1 ± 1	10 ± 0.8	14 ± 1.4	7.7 ± 0.5	23.3 ± 5.2
Act 9	13.6 ± 0.9	12 ± 0.9	12 ± 0.9	12 ± 0.9	8 ± 1.2	10.8 ± 0.9	16.6 ± 3.4
Act 10	10.8 ± 0.7	8.1 ± 0.4	6 ± 0.4	11.1 ± 1	13 ± 2.4	11.9 ± 1.2	14.2 ± 1.6
Act 11	10.3 ± 0.7	10 ± 0.8	10 ± 0.9	10 ± 0.9	5.8 ± 1.8	12.7 ± 1.0	8.7 ± 1.3
Act 12	19.4 ± 1.6	20.2 ± 1	24 ± 0.5	29 ± 2.7	21 ± 4.5	22.50 ± 4	20.0 ± 2.5
Act 13	14.8 ± 0.8	18 ± 0.8	19 ± 0.8	15 ± 0.8	25 ± 1.2	19.8 ± 0.8	22.5 ± 1.8
Act 14	8.9 ± 0.5	13.4 ± 1	16 ± 1.3	17 ± 0.3	11 ± 3.7	15.6 ± 0.9	9.7 ± 3.7
Act 15	18.2 ± 1.5	24 ± 2.1	15 ± 5.1	29 ± 0.9	26 ± 4.1	27.2 ± 3.2	26.3 ± 3.7
Act 16	13 ± 0.6	17 ± 0.8	18 ± 0.9	21.4 ± 1	23 ± 1.1	22.8 ± 1.1	27.3 ± 1.7
Act 17	8.9 ± 0.6	10 ± 0.6	10 ± 0.6	10 ± 0.6	14 ± 1.1	9.70 ± 0.7	15.1 ± 1.4
Act 18	10.5 ± 0.8	12.5 ± 1	13 ± 1.1	14.5 ± 1	15 ± 0.8	17.1 ± 1.4	14 ± 0.8
Act 19	20.4 ± 1.4	26 ± 2.3	24 ± 0.6	28 ± 0.2	29 ± 2.8	26.0 ± 5.5	19.2 ± 2
Act 20	15.3 ± 0.7	16 ± 0.7	17 ± 0.7	18.0.7	14 ± 0.7	25.6 ± 0.6	12.8 ± 0.7
Act 21	16.9 ± 1.9	18 ± 0.7	20 ± 0.6	24.2 ± 1	22 ± 2.6	21.7 ± 1.1	23.5 ± 1.1

**Table 4:** The antioxidant phenolic, flavonoids and tocopherols content as well as pigment profile of 21 bacterial isolates. Data are represented by the means of at least 3 replicates ± standard deviations (SD).

Isolates	Total Flavonoids	Total Phenols	Tocopherols	Lutine	Alpha-Carotene	β-Cryptoxanthin	β-Carotene	Gamma-Carotene	Lycopene	Carotenoids
Act 1	4.6 ± 0.3	35.3 ± 2.4	0.3 ± 0	0.3 ± 0.02	0.42 ± 0.03	0.24 ± 0.02	0.27 ± 0.02	0.06 ± 0	0.08 ± 0.01	0.93 ± 0.09
Act 2	8 ± 0.6	48.6 ± 3.6	0.5 ± 0	0.5 ± 0.04	0.63 ± 0.05	0.28 ± 0.02	0.28 ± 0.02	0.1 ± 0.01	0.13 ± 0.01	1.3 ± 0.12
Act 3	6.7 ± 0.4	31.4 ± 1.7	ND ± ND	0.3 ± 0.01	0.34 ± 0.01	0.3 ± 0.02	0.31 ± 0.02	0.06 ± 0	0.07 ± 0	1.07 ± 0.05
Act 4	5.4 ± 0.3	35.6 ± 3.3	ND ± ND	0.2 ± 0.02	0.23 ± 0.03	0.38 ± 0.06	0.42 ± 0.06	0.1 ± 0.02	0.1 ± 0.02	0.59 ± 0.15
Act 5	5.8 ± 0.4	29.9 ± 1.6	0.1 ± 0	0.1 ± 0	0.14 ± 0	0.27 ± 0.01	0.29 ± 0.01	0.29 ± 0.02	0.23 ± 0.02	0.61 ± 0.02
Act 6	6.9 ± 0.5	29.6 ± 2.2	0.3 ± 0	0.1 ± 0.01	0.18 ± 0.02	0.35 ± 0.03	0.47 ± 0.05	0.14 ± 0.01	0.12 ± 0.01	0.77 ± 0.06
Act 7	7.4 ± 0.6	31.1 ± 2	ND ± ND	0.1 ± 0	0.12 ± 0.01	0.21 ± 0.01	0.23 ± 0.01	0.21 ± 0.02	0.38 ± 0.04	0.8 ± 0.05
Act 8	9.5 ± 0.8	49.4 ± 4.1	ND ± ND	0.2 ± 0.02	0.23 ± 0.02	0.17 ± 0.03	0.18 ± 0.01	0.09 ± 0	0.16 ± 0.01	0.48 ± 0.03
Act 9	11.2 ± 0.9	33.6 ± 1.7	0.4 ± 0	0.2 ± 0.01	0.27 ± 0.02	0.14 ± 0.01	0.12 ± 0.01	0.13 ± 0.01	0.61 ± 0.07	0.97 ± 0.08
Act 10	10.4 ± 0.7	20.7 ± 1.3	ND ± ND	0.2 ± 0.01	0.17 ± 0.01	0.2 ± 0.02	0.27 ± 0.04	0.09 ± 0.01	0.22 ± 0.01	0.82 ± 0.04
Act 11	8.8 ± 0.6	24.5 ± 1.8	0.3 ± 0	0.2 ± 0.01	0.21 ± 0.02	0.23 ± 0.02	0.19 ± 0.02	0.04 ± 0	0.04 ± 0.01	0.99 ± 0.08
Act 12	11.5 ± 0.9	48.2 ± 3.9	0.5 ± 0	0.2 ± 0.02	0.33 ± 0.02	0.86 ± 0.08	0.85 ± 0.09	0.1 ± 0.01	0.08 ± 0.01	1.67 ± 0.13
Act 13	9.7 ± 0.6	39.3 ± 2.1	0.2 ± 0	0.3 ± 0.03	0.44 ± 0.03	0.5 ± 0.02	0.56 ± 0.02	0.08 ± 0	0.1 ± 0.01	1.06 ± 0.05
Act 14	3.9 ± 0.1	35.3 ± 2.5	ND ± ND	0.2 ± 0.01	0.35 ± 0.02	0.14 ± 0	0.58 ± 0	0.24 ± 0.02	0.19 ± 0.03	1 ± 0.08
Act 15	9.9 ± 0.9	48.5 ± 3.6	0.5 ± 0	0.3 ± 0.03	0.45 ± 0.04	0.17 ± 0.01	0.38 ± 0.02	0.19 ± 0.01	0.19 ± 0.01	1.58 ± 0.12
Act 16	8.7 ± 0.5	33.4 ± 1.7	0.3 ± 0	0.2 ± 0.01	0.32 ± 0.02	0.4 ± 0.03	0.44 ± 0.04	0.09 ± 0	0.09 ± 0.01	1.24 ± 0.07
Act 17	4.8 ± 0.3	31.9 ± 2.4	ND ± ND	0.1 ± 0.01	0.16 ± 0.01	0.36 ± 0.03	0.43 ± 0.02	0.05 ± 0	0.05 ± 0	0.53 ± 0.02
Act 18	5.1 ± 0.4	40.1 ± 3	0.2 ± 0	0.2 ± 0.02	0.32 ± 0.03	0.18 ± 0.01	0.2 ± 0.01	0.08 ± 0.01	0.09 ± 0.02	0.49 ± 0.04
Act 19	9.8 ± 0.8	59.8 ± 4.5	0.5 ± 0	0.2 ± 0.01	0.23 ± 0.01	0.27 ± 0	0.82 ± 0	0.14 ± 0.01	0.08 ± 0.01	1.29 ± 0.12
Act 20	8.3 ± 0.5	38.6 ± 2.1	ND ± ND	0.1 ± 0	0.08 ± 0.01	0.17 ± 0.01	0.44 ± 0.01	0.15 ± 0.01	0.38 ± 0.03	1.14 ± 0.06
Act 21	7.7 ± 0.5	51.7 ± 5	0.4 ± 0.1	0.1 ± 0.02	0.2 ± 0.03	0.15 ± 0.03	0.17 ± 0.02	0.29 ± 0.09	0.15 ± 0.01	0.84 ± 0.25

**Table 5:** The phenolic profile of five most biologically active *Streptomyces* isolates (Act 2, Act 12, Act 15, Act 19 and Act 21). Data are represented by the means of at least 3 replicates and error bars represent standard deviations.

Phenolic Compounds	Act 2	Act 12	Act 15	Act 19	Act 21
Caffeic acid	0.79 ± 0.05	0.75 ± 0.19	0.77 ± 0.06	1.25 ± 0.09	1.05 ± 0.22
Ferulic ACID	5.5 ± 0.23	5.55 ± 0.23	5.15 ± 0.38	5.43 ± 0.34	6.68 ± 0.32
Protocatechuic acid	10.2 ± 0.41	20 ± 2.49	9.26 ± 0.69	16.12 ± 0.65	21.09 ± 1.61
Catechin	4.4 ± 0.18	6.67 ± 0.13	4.01 ± 0.3	7.02 ± 0.28	8.78 ± 0.13
Gallic acid	14.4 ± 0.58	21.8 ± 1.24	13.11 ± 0.97	23 ± 0.93	24.55 ± 1.92
p-Coumaric acid	12.1 ± 0.49	9.04 ± 0.37	11.02 ± 0.82	9.15 ± 0.7	20.24 ± 1.58
Resorcinol	ND	0.09 ± 0	0.11 ± 0.01	ND	0.21 ± 0.02
Chlorogenic acid	0.74 ± 0.03	1.53 ± 0.34	ND	1.18 ± 0.05	1.76 ± 0.2
Sinapic acid	ND	8.53 ± 0.21	ND	2.3 ± 0.09	11.95 ± 0.94
Quercetin	10.3 ± 0.42	7.72 ± 0.31	9.41 ± 0.7	ND	7.31 ± 0.09
Quercetrin	1.15 ± 0.05	0.86 ± 0.03	1.05 ± 0.08	1.83 ± 0.07	3.32 ± 0.26
Luteolin	0.34 ± 0.01	0.92 ± 0.41	ND	0.85 ± 0.03	ND
Apigenin	ND	1.3 ± 0.2	ND	1.89 ± 0.27	ND
Isoquercetrin	ND	1.2 ± 0.05	1.47 ± 0.11	1.35 ± 0.12	3.27 ± 0.07
Rutin	4.98 ± 0.36	5.41 ± 0.22	6.59 ± 0.49	6.46 ± 1.02	18.57 ± 1.45
Ellagic acid	2.89 ± 0.12	2.16 ± 0.09	2.63 ± 0.19	3 ± 0.14	7.45 ± 0.58
Velutin	ND	2.61 ± 0.11	ND	ND	4.1 ± 0.06
Naringenin	ND	0.04 ± 0.0	0.04 ± 0.003	0.11 ± 0.004	0.11 ± 0.009
Genistein	ND	0.03 ± 0.0	0.03 ± 0.002	0.08 ± 0.003	0.09 ± 0.007
Daidzein	ND	0.02 ± 0.03	0.02 ± 0.001	0.06 ± 0.002	ND
Fisetin	0.012 ± 0	ND	ND	ND	0.031 ± 0.001
O-hydroxydaidzein	ND	0.01 ± 0	0.06 ± 0.005	0.03 ± 0.001	0.06 ± 0.003

## Data Availability

Not applicable.
